# The Bladder EpiCheck Test as a Non-Invasive Tool Based on the Identification of DNA Methylation in Bladder Cancer Cells in the Urine: A Review of Published Evidence

**DOI:** 10.3390/ijms21186542

**Published:** 2020-09-08

**Authors:** Mariangela Mancini, Marialaura Righetto, Sara Zumerle, Monica Montopoli, Filiberto Zattoni

**Affiliations:** 1 Department of Surgical, Oncological and Gastroenterological Science, Urological Clinic, University of Padua, 35128 Padua, Italy; marialaura.righetto@gmail.com (M.R.); filiberto.zattoni@unipd.it (F.Z.); 2 Department of Medicine, Veneto Institute of Molecular Medicine, University of Padua, 35128 Padua, Italy; sara@zumerle.net; 3 Department of Pharmaceutical and Pharmacological Sciences, University of Padua, 35128 Padua, Italy; monica.montopoli@unipd.it

**Keywords:** bladder cancer, DNA methylation, biomarkers

## Abstract

Recently, there has been a great effort to develop tests based on non-invasive urinary biomarkers (NMIBCs). These tests are based on the fact that NMIBCs are heterogeneous at the molecular level and can be divided into different molecular groups useful to predict prognosis and response to treatment. The assessment of epigenetic alterations, such as DNA methylation, represents a promising cancer biomarker. DNA methylation is an epigenetic modification that affects gene expression without modifying the DNA sequence. Several studies have highlighted the presence of methylated loci in the context of bladder cancer, indicating its potential application as a diagnostic and prognostic biomarker. One of the novel assays based on a DNA methylation profile, the Bladder EpiCheck, analyzes DNA from spontaneous urine, detecting disease-specific DNA methylation patterns in bladder cancer patients. This test, due to its non-invasive nature and highly promising performance could, in future, become an invaluable tool in the follow-up of bladder cancer patients. Potential new applications could include diagnosis and surveillance of upper-tract disease, for the replacement of invasive testing and ureteroscopy.

## 1. Introduction

### 1.1. Background

Non-muscle invasive bladder cancer (NMIBC) has high recurrence rates up to 60% within the first year of diagnosis [1]. Most patients will experience numerous recurrences during follow-up, while some others will experience progression to a muscle-invasive disease. The factors associated with the recurrence of NMIBC are grade (low-grade or high-grade disease), number and size of tumors, number of previous recurrences, tumor stage, and presence of concurrent carcinoma in situ [2]. Moreover, NMIBC is a dangerous chronic disease, leading to a substantial burden on patients and healthcare systems [3]. Surveillance of NMIBC to early-identify recurrence, progression, or localization in different areas of the urinary tract is crucial and is currently based on repeated cystoscopies and urine cytologies [1]. Both of these exams present some issues: cystoscopy is an invasive and expensive procedure that could potentially lead to life-threating morbidities; urine cytology, on the other hand, has low sensitivity, low diagnostic accuracy and is operator-dependent, despite being a less expensive exam and having a high-specificity for high-grade NMIBC.

Recently, therefore, there has been a great effort to develop tests based on non-invasive urinary biomarkers [4,5]. These tests are based on the fact that NMIBCs are heterogeneous at the molecular level and can be divided into different molecular groups useful to predict prognosis and response to treatment [6]. The assessment of epigenetic alterations, such as DNA methylation, represents a promising cancer biomarker. DNA methylation is an epigenetic modification that affects gene expression without modifying the DNA sequence. Several studies have highlighted the presence of methylated loci in the context of bladder cancer, indicating its potential application as a diagnostic and prognostic biomarker [7,8]. One of the novel assays based on a DNA methylation profile, the Bladder EpiCheck (BE), analyzes DNA from spontaneous urine, thereby detecting disease-specific DNA methylation patterns in bladder cancer patients.

### 1.2. Accuracy of a Diagnostic Test

The ideal urinary biomarker to use in clinical practice should have good cost-benefits and good sensitivity and specificity. Moreover, it should be simple and quick to interpret, with a good diagnostic accuracy and inexpensive. The urinary markers developed so far do not have high specificity and sensitivity, and they have a lot of false positives and false negatives, as compared to a reference threshold. Consequently, these markers increasingly have a reduced positive predictive value (PPV) and negative predictive value (NPV), the majority having sensitivity and specificity ranging between 50–80% [9].

We need a test with high NPV for all non-muscle invasive bladder cancers (NMIBCs), especially for high-grade NMIBCs. NPV is a point of reference because it could avoid unnecessary cystoscopies.

### 1.3. Bladder EpiCheck Test

The Bladder EpiCheck™ (Nucleix, Rehovot, Israel) test is a non-invasive assay recently developed for the surveillance of bladder cancer recurrence. The analysis is based on the detection of the DNA methylation status of 15 genomic loci which are associated with a high prevalence of DNA methylation in bladder cancer cells.

Ten milliliters of urine represent the minimal amount of urine to perform the assay reliably. Genomic DNA is extracted from cells obtained by urine centrifugation and digested using a methylation-sensitive restriction enzyme which cleaves DNA at specific sites only in the absence of methylation. The methylation status of the 15 biomarkers is then assessed by real-time polymerase chain reaction (PCR) and analyzed using a specific software, thus providing a numerical value called an EpiScore™. Samples with an EpiScore™ superior to 60, on a 0–100 range, are considered positive for bladder cancer.

## 2. Evidence Acquisition

### 2.1. Search Strategies, Selection of Studies, and Data Extraction

This is a narrative review. Databases including MEDLINE, Embase and the Cochrane Library were systematically searched. We used different combinations of the keywords “non-muscle-invasive bladder cancer”, “urinary biomarker”, “Bladder EpiCheck”, and “DNA methylation test”, and we included results up to 31 May 2020. No language restriction was applied. All studies describing the application of the Bladder EpiCheck test (BE) to patients with NMIBC were included, without exclusion criteria. We excluded from this review articles about BE for the surveillance of the upper urinary tract urothelial carcinoma. Full-text articles and non-full-text articles only (i.e., conference abstracts) published on BE were included in this review. The authors screened articles independently.

### 2.2. Types of Participants Included

Concerning all studies, the study population was limited to patients who were in surveillance for histologically proven NMIBC of any grade (carcinoma in situ – CIS, non-invasive papillary carcinoma - Ta, carcinoma that invades subepithelial connective tissue - T1; no regional lymph node metastasis clinically identified - cN0, no distant metastasis clinically identified - cM0) according to the TNM (tumor – nodes – metastasis) staging system (all versions). No gender limit was considered. Two studies [10,11] included only patients with NMIBC resected within the previous 12 months.

Data from patients with a first diagnosis of bladder cancer, patients with muscle-invasive bladder cancer, and patients with non-localized disease (with clinically positive regional lymph nodes or distant metastasis - cN+, cM+) were excluded in all studies.

Patients who were having adjuvant intravesical therapy and surveillance cystoscopies at 3–6-month intervals also were included in the studies.

### 2.3. Types of Intervention and Outcome Measures Included

BE was performed on patient urine samples collected before standard-of-care cystoscopy.

The primary outcomes were the sensitivity and specificity of BE during surveillance for NMIBC. Both sensitivity and specificity of the BE were compared to the cystoscopy, cytology, and histology results for a reference standard. The secondary outcome was the EpiScore continuous measure for each patient.

### 2.4. Data Analysis

Regarding each paper, we collected information on study design, inclusion and exclusion criteria, patient characteristics, and outcome measures. We used descriptive statistics to summarize baseline data and to plan a narrative synthesis of the data.

## 3. Evidence Synthesis

### 3.1. Quantity of Evidence Identified and Characteristics of the Studies Included

Overall, 11 records were identified through database searching, and eight were screened after removal of duplicates and of a study on upper tract urothelial carcinoma surveillance. Considering these, five articles were eligible for full-text screening, and three conference abstracts were assessed for eligibility.

Finally, eight studies recruiting a total of 1993 patients met the inclusion criteria. All the studies were prospective, blinded, single-arm cohort studies, with five regarding multicentric tumors [10,11,12,13,14] and three regarding monocentric tumors [15,16,17].

One study was a secondary external independent analysis of the results of a previous one [11], a second study was an updated version of a previous one, with additional patients [14], and a third study was a comparative study on two types of Real-Time Polymerase Chain Reaction-Based (RT-PCR) urinary markers, based on the population analyzed in a previous study [13].

### 3.2. Reference Standard Definition and Follow-Ups

Regarding all studies, the sensitivity and specificity of the BE were compared to a prespecified reference standard, defined as the result of cystoscopy, cytology, and/or histology. The definition of the reference standard and, consequently, of its result, is a key point in the evaluation of the positivity or negativity of the BE. Concerning the case of a positive cystoscopy (suspicious for recurrence), histology was performed, either an endoscopic cold-cup biopsy or a transurethral resection (TURB). Regarding the case of a positive/suspicious wash cytology, subsequent endoscopic mapping biopsies were planned.

To define a ‘negative’ patient, the white-light cystoscopy, wash cytology, and histology must be negative. Particularly, for cytological specimen classification, all studies used the Paris System for Reporting Urinary Cytology. During three of the studies [10,11,17] the wash cytology was considered ‘negative’ when the result was ‘negative for high-grade urothelial carcinoma (HGUC)’ and ‘atypical urothelial cells’, whereas ‘suspicious for HGUC’, ‘HGUC’, and ‘low-grade (LG) intraepithelial neoplasia’ were clustered as ‘positive’. Concerning the case of positive spontaneous or wash cytology with negative cystoscopy and/or histology, the reference standard was evaluated at the subsequent follow-up cystoscopy with multiple random biopsies or targeted biopsies on a suspicious area [17]. Concurrently, in the study by Pierconti et al. [16], patients with a cytological diagnosis of ‘atypical urothelial cells’ also were considered ‘positive’ and underwent cystoscopy within three months, with multiple random biopsies in the case of a negative cystoscopy.

Notably, Witjes at al., [10] used more rigid parameters in the reference standard definition, considering the pathology specimen as the key determinant. When the patient had a positive pathology, the sample was considered ‘positive’; when the pathology was negative, but the cytology was positive, the sample was considered inconclusive, and excluded from the final analysis. Similarly, samples with positive or equivocal cystoscopic or cytologic results without a subsequent confirmatory pathology also were classified as inconclusive and excluded from the final analysis.

Patient follow-ups after BE varied from six months [17] to 12 months [11,12,13,14,15,16,17]. Follow-up data was not available from one study [10].

### 3.3. Statistical Methods

All studies conducted a sensitivity analysis to check the performance of BE in the detection of low and high-grade bladder cancers. The discrimination of BE was assessed graphically with the receiver operating characteristic (ROC) curve and the area under the curve (AUC), an immediate measure for the EpiScore continuous variable. Additional statistics evaluating the accuracy of the BE included the positive and negative predictive value (PPV and NPV, respectively), the positive and negative likelihood ratio (PLR and NLR, respectively), and the receiver-operating curve (ROC). The area under the ROC curve (AUC) was a summary measure for the EpiScore continuous value.

Unlike the other studies, D’Andrea et al. [11] evaluated the association between the BE result and bladder cancer recurrence by generating two multivariate nomograms for the prediction of the presence of any-grade and high-grade bladder cancer, respectively. Moreover, the authors performed a decision-curve analysis (DCA) to evaluate the utility of BE on decision-making in routine clinical practice.

### 3.4. Results: Sensitivity, Specificity, and Predictive Values Analysis

The validation study by Wasserstrom et al. [15] applied BE on a sample population of 222 patients. Histological diagnosis of bladder cancer was possible for 40 patients (18%), and BE correctly identified 36 malignancies, with an overall sensitivity of 90%. Notably, BE sensitivity changed based on staging and grading, with a higher sensitivity in higher stages (81%, 100%, 100%, 91%, in Ta, T1, T2 (tumor invading muscularis) and CIS, respectively), and in higher grades (84% in low-grade, and 95% in high-grade tumors). The overall BE specificity was 83%, correctly diagnosing 151/182 cancer-free patients, and the NPV was 97%.

Conversely, considering wash cytology as the reference standard, BE showed a greater sensitivity (90% versus 38%, in low and high-grade tumors), but a lower specificity (83% versus 96%).

This means that BE detected all cancers that also were detected by cytology, and that all cancers missed by BE also were missed by cytology. Finally, the authors highlighted that high histological grading was significantly associated with a higher mean EpiScore score, than was low grading (85 versus 69, respectively; *p* = 0.006).

Witjes et al. [10] tested BE on 440 patients, performing statistical analysis on 353 of them (87 subjects excluded due to inconclusive diagnosis according to the reference standard, no BE results, or both). Forty-four out of three hundred and fifty-three patients (12.5%) were ‘positive’ according to the reference standard.

The overall sensitivity was 68% (30/44) and the overall specificity was 88% (272/309). The test performance did not vary with age, gender, smoking habits, occupational exposure, and treatment for recent recurrence (stopped or ongoing). Like the previous study, BE sensitivity varied according to the staging and grading, with a higher sensitivity in higher stages (52%, 100%, 100% for Ta, T1 and CIS, respectively) and grades (40% in low-grade, and 89% in high-grade tumors). NPV was high for the entire patient cohort (95%). Notably, BE could exclude the presence of high-grade NMIBCs with a NPV of 99%, and, conversely, it could detect the presence of high-grade NMIBCs with a sensitivity of 92%. The AUC was 0.82 for all tumors (including low-grade Ta), and 0.94 when excluding low-grade recurrences.

Lozano et al. [14] recently updated the results of the previous study, with the addition of 382 patients, 304 of whom were recorded for results analysis (final cohort: 657 patients). Eighty patients were ‘positive’ as compared to the reference standard. The results were similar between the first and the second analyses (overall sensitivity: 62.5%; sensitivity when excluding low-grade NMIBCs: 86%; specificity: 86%). NPV was 94%. Notably, BE outperformed cytology in all categories (all-grade, low-grade, and high-grade NMIBCs). Moreover, in high-grade NMIBCs, BE showed a higher sensitivity than cystoscopy (86% versus 73%; *p* = 0.113), although not significant. Conversely, BE sensitivity in the low-grade disease was significantly lower than cystoscopy (33% versus 94%; *p* < 0.0001).

A secondary external independent analysis of the study by Witjes et al. [11] tested BE performance on 357 patients, with 49/357 (13.7%) intravesical disease recurrences. The study showed an overall sensitivity and specificity of 67% and 88%, respectively. The sensitivity rose to 89% for the detection of high-grade NMIBCs. NPV was 94% for any-grade NMIBCs, and 99% for high-grade NMIBCs.

Considering univariable logistic regression analyses, a one-point increase in the EpiScore corresponded to a 4% increase in the risk of any-grade bladder cancer, and to an 8% increase in the risk of high-grade NMIBC. Using multivariable logistic regression analysis, a positive BE result was independently associated with the presence of any-grade (odds ratio, OR = 18.1, 95% CI 8.66–40.2; *p* < 0.001) and high-grade NMIBC (OR = 78.3, 95% CI 19.2–547; *p* < 0.001).

Finally, using explorative analysis, the performance of BE, evaluated by the AUC, was not affected by epidemiological features (age, gender, time from last recurrence, ongoing intravesical therapy, pathological stage and grade), and the addition of BE to these clinical variables significantly improved their AUC by 16% for the prediction of any-grade bladder cancer and 22% for the prediction of high-grade NMIBCs (BE AUC: 86% and 96% for any-grade and high-grade NMIBC, respectively).

During the study by Trenti et al. [12], BE was tested on 243 patients, with 215 evaluable results (28 test failures excluded from the final analysis). Sixty-nine (32%) patients had an NMIBC recurrence. The overall BE sensitivity was 62%, which rose to 83% for high-grade NMIBCs. The overall BE specificity was 86%, and the NPV was 83%. The diagnostic efficacy of BE was good, with an AUC of 0.785. The lower accuracy and lower NPV of BE in this study was probably due to the higher prevalence of NMIBC recurrence.

A subsequent study by the same authors [13] compared BE to another Real-Time Polymerase Chain Reaction-Based urinary marker (Xpert Bladder Cancer Monitor [18]), which measures the level of five target messenger RNAs, upregulated in urine samples from patients with bladder cancer. The study enrolled 487 patients, 55 of whom had an invalid BE (total population: 432 patients). Twenty-one percent of the patients had an NMIBC recurrence. The test showed an overall sensitivity of 64% (66% for the Xpert Bladder Cancer Monitor test), which increased to 79% in high-grade NMIBCs (57% for low-grade NMIBCs). The overall specificity was 82% for the BE. The NPV was almost the same for both BE and Xpert Bladder Cancer Monitor (about 89% for both). BE demonstrated a high diagnostic efficacy, with an AUC of 74% (95% CI, 67.2–80.5%).

A study by Righetto et al. [17] on 88 patients and 72 BE results considered for the statistical analysis, showed a prevalence of recurrent NMIBC in 17.3% of the study population. The sensitivity of BE in all-grade NMIBCs was 76%, which rose to 100% (95% CI, 75.5–100%) for high-grade NMIBCs. The overall specificity of the BE was 90%, the NPV was 97% in all-grade NMIBCs, and 100% in high-grade NMIBCs.

Clinical data regarding the BE performance among the published studies are summarized in Table 1.

### 3.5. Results: BE and Cytology

Few studies have compared the sensitivity, PPV and NPV for both cytology and BE, and only one evaluated the diagnostic accuracy of the combination of the two tests [12].

Seen in all studies, the sensitivity of cytology was lower than the BE sensitivity (Table 2), with percentages ranging from 27% [13] to 38% maximum [15]. Particularly, the cytology sensitivity was markedly low in low-grade NMIBCs (0–13% across studies), and increased, while remaining lower than BE sensitivity, in high-grade NMIBCs (50–67% across studies). Notably, the NPV of the BE was higher than cytology NPV in all studies. Thus, the high sensitivity and NPV of BE could be used as an additional tool to improve the sensitivity of cytology.

The EpiScore, as a continuous variable, also could stratify differently according to different results of cytology. This data suggests that different cytological categories also carry a distinct methylation (thus, molecular) pattern.

Pierconti et al. [16] analyzed the EpiScore results of 374 patients with high-grade NMIBC (268 T1, 106 CIS) according to different cytological results (negative for high-grade urothelial carcinoma, NHGUC; atypical urothelial cells, AUC; suspicious for high-grade urothelial carcinoma, SHGUC; high-grade urothelial carcinoma, HGUC; low-grade urothelial neoplasia, LGUC; unsatisfactory/non-diagnostic), finding that differences between cytological categories also could be based on molecular disparities. Actually, the EpiScore increased from NHGUC to HGUC; an EpiScore <60 correlated with NHGUC (OR = 3.9, 95% CI 1.9–8.1, *p* = 0.0003), while an EpiScore ≥60 correlated with SHGUC (OR = 3.8, 95% CI 1.6–8.9; *p* = 0.0031) and HGUC (OR = 3.9, 95% CI 1.6–9.5; *p* = 0.0027). Moreover, during a one year follow-up, the EpiScore had a sensitivity of 100%, a specificity of 89.9%, and a NPV of 100% in the NHGUC group while, in the HGUC group, the EpiScore reached a sensitivity of 98%, a specificity of 100%, and a NPV of 86%.

A study by Trenti et al. [12] evaluated the possibility of combining BE and cytology results with the aim of increasing the diagnostic accuracy. The authors showed that the overall sensitivity of cytology (33%) could significantly rise in combination with BE (67% for the two tests combined). Particularly, the sensitivity for low-grade NMIBCs varied from 8% of cytology to 46% of BE, while their combination yielded an overall sensitivity of 56%. Conversely, the sensitivity for high-risk NMIBCs was 67% for cytology and 83% for BE alone, whereas the combination of the two tests obtained an overall sensitivity of 90%.

The authors did not find a significant advantage in the combination of the two tests regarding PPV and NPV. The PPV was higher for cytology than for BE (92% versus 68%), while the NPV was similar for both (75.8% for cytology, 82.9% for BE). The combination of the two tests failed to reach a significant improvement in PPV and NPV (overall PPV: 69%, overall NPV: 84.5%).

To conclude, the combination of both cytology and BE for the surveillance of patients with NMIBC could increase the sensitivity of the individual tests, especially in high-grade NMIBCs. Consequently, the combination of the two tests could potentially reduce the number and frequency of cystoscopies during follow-ups of patients with NMIBCs, especially for those with low-risk disease. However, we must consider that BE needs a dedicated and equipped laboratory to perform real time-PCR.

### 3.6. Results: Decision-Curve Analysis (DCA)

The study by D’Andrea et al. [11] proposed two nomograms for the prediction of any-grade bladder cancer and high-risk bladder cancer during the follow-up of patients with NMIBC. The authors assessed the BE clinical utility and its impact on decision-making using decision-curve analysis (DCA), showing that current predictors of NMIBC recurrence (i.e., last stage and grade) performed no better than cystoscopy in every patient (which meant that all patients with NMIBC needed cystoscopy during their follow-up). Conversely, the use of BE for deciding whether to perform a cystoscopy or not during the follow-up of these patients offered a net benefit of BE relative to evaluating all patients with cystoscopy. This net benefit was maintained across threshold probabilities between 10–60% for the presence of any-grade bladder cancer, and 5–55% for high-grade bladder cancer. All decisions are currently based on clinical and epidemiological predictors, within these ranges. This data is fundamental during the decision-making process. BE could reduce the number of unnecessary cystoscopies and cytologies, without missing any cancer, in patients with a risk of recurrence >10% for any-grade NMIBCs and > 5% for high-grade NMIBCs.

### 3.7. BE and Other Bladder Tumor Markers

Several molecular markers for both the diagnosis and follow-up of NMIBC are Food and Drug Administration approved. Particularly, for the follow-up of NMIBC, a non-invasive urine test with the best clinical utility should avoid unnecessary cystoscopies. Furthermore, such a urinary marker should have a high NPV—able to predict the absence of bladder cancer before cystoscopy.

Comparing BE and other markers used for the follow-up of patients with NMIBC [19], BE shows a higher sensitivity than a bladder tumor antigen stat test (BTA stat test; sensitivity range: 57–83%), an ImmunoCyt (sensitivity range: 50–100%), a fluorescence in situ hybridization (FISH–Urovysion; sensitivity: 70%), and a nuclear matrix protein 22 (NMP 22) test (sensitivity range: 47–100%).

However, there are two major points that play against BE as compared to other markers. First, BE requires a dedicated technician and a molecular laboratory to perform real time-PCR. Second, BE is not as cheap as the other tests.

## 4. Conclusions

BE has a high diagnostic accuracy and shows a high performance for surveillance of patients with NMIBC. Particularly, BE expresses the maximum diagnostic power in patients with low-grade bladder cancer. Regarding patients with a history of high-grade NMIBC, BE could be useful to increase the interval between cystoscopies, considering its very high NPV and high sensitivity.

BE showed, in several studies, a high NPV (see Table 2). It also showed that in positive cases it is able to select subsets of bladder cancer with negative cystoscopy and positive/negative cytology which seem to present DNA methylation changes. These ‘false-positive’ BE could represent a genetic high-risk population that could relapse in the near future and deserves close clinical attention. BE negative cases, on the other hand, could represent a population of high-risk bladder cancers with a more stable methylation pattern, which could present an inferior risk of progression.

## Figures and Tables

**Table 1:** Performance of Bladder Epicheck test among studies.

Studies	BE Results Considered for the Statistical Analysis	NMIBC Recurrences Prevalence	Sensitivity		Specificity	PPV	NPV	
			All Grades	non-LG			All Grades	non-LG
**Wasserstrom et al. (2016)**	222	18%	90%	95%	83%	-	97%	-
**Witjes et al. (2018)**	353	12.5%	68.2%	88.9%	88%	44.8%	95.1%	99.3%
**Lozano et al. (2019)**	657	12.2%	62.5%	86.4%	85.8%	-	94.3%	98.8%
**D’Andrea et al. (2019)**	357	13.7%	67.3%	89%	88%	47%	94%	99.3%
**Trenti et al. (2019)**	215	32.1%	62.3%	83.3%	86.3%	68.2%	82.9%	-
**Righetto et al. (2019)**	72	29.2%	76.2%	100%	90.2%	76.2%	97.4%	100%
**Trenti et al. (2020)**	432	21.3%	64.1%	78.9%	82.1%	49.1%	89.4%	-

NMIBC: non-muscle-invasive bladder cancer; BE: Bladder EpiCheck test; PPV: positive predictive value; NPV: negative predictive value; LG: low-grade bladder cancers.

**Table 2:** Sensitivity, Specificity, PPV, and NPV of Cytology, Bladder EpiCheck, and the combination of the two tests between studies.

Studies	Cytology	EpiCheck	EpiCheck + Cytology
	%, (95% CI)	%, (95% CI)	%, (95% CI)
**Wasserstrom et al. (2016)**			
Sensitivity	38%	90%	
Specificity	96%	83%	
**Trenti et al. (2019)**			
Sensitivity	33.3 (22.4–45.7)	62.3 (32.6–59.7)	66.7 (54.3–77.5)
*Low-grade NMIBC*	*7.7 (1.6*–*20.9)*	*46.1 (30.1*–*62.8)*	*48.7 (32.4*–*65.2)*
*High-grade NMIBC*	*66.7 (47.2*–*82.7)*	*83.3 (65.3*–*94.4)*	*90 (73.5*–*97.9)*
Specificity	98.6 (95.1–99.8)	86.3 (79.6–91.4)	85.6 (78.8–90.9)
PPV	92.0 (73.6–97.9)	68.2 (57.9–77.1)	68.6 (58.8–77.1)
NPV	75.8 (72.6–78.7)	78.6 (72.5–83.9)	84.5 (79.4–84.7)
**Righetto et al. (2019)**			
Sensitivity	35.7 (12.8–64.8)	76.2 (52.8–91.8)	-
*Low-grade NMIBC*	*0 (0*–*60.2)*	*37.5 (85*–*75.5)*	
*High-grade NMIBC*	*50 (18.7*–*81.3)*	*100 (75.3*–*100)*	-
Specificity	96.8 (83.3–99.9)	90.2 (78.6–96.7)	-
Accuracy	77.8% (62.9–88.8)	86.1% (75.9–93.1)	
*Low-grade NMIBC*	*85.7 (69.7*–*95.2)*	*83.0 (71.0*–*91.6)*	
*High-grade NMIBC*	*85.4 (70.8*–*94.4)*	*92.2 (82.7*–*96.7)*	
**Trenti et al. (2020)**			
Sensitivity	27.2 (18.4–37.4)	64.1 (53.5–73.9)	-
*Low-grade NMIBC*	*12.9 (5.4*–*24.9)*	*53.7 (39.6*–*67.4)*	
*High-grade NMIBC*	*47.4 (30.9*–*64.2)*	*78.9 (62.7*–*90.4)*	
Specificity	98.8 (97.0–99.7)	82.1 (77.6–85.9)	-
PPV	86.2 (69.0–94.6)	49.2 (42.4–55.9)	-
NPV	83.6 (79.7–86.9)	89.4 (86.5–91.8)	-

PPV: positive predictive value; NPV: negative predictive value; CI: confidence interval.
